# Febrile neutropenia management in pediatric cancer patients at Ethiopian Tertiary Care Teaching Hospital

**DOI:** 10.1186/s13104-019-4569-5

**Published:** 2019-08-20

**Authors:** Husnia Bedewi Mohammed, Malede Berihun Yismaw, Atalay Mulu Fentie, Tamrat Assefa Tadesse

**Affiliations:** School of Pharmacy, College of Health Sciences, Addis Ababa University, P.O.Box:9086, Addis Ababa, Ethiopia

**Keywords:** Febrile neutropenia, Cancer in pediatrics, Tikur Anbessa Specialized Hospital, Ethiopia

## Abstract

**Objective:**

This study aimed at assessing the management practice of febrile neutropenia (FN) in pediatric cancer patients at Tikur Anbessa Specialized Hospital (TASH), Ethiopia by reviewing patients’ charts from 135 participants retrospectively. Data was entered into Epi-info 7 and exported to SPSS 20 for analysis.

**Results:**

Empiric antibiotics therapy (EAT) was given to all patients in which ceftriaxone with gentamycin constituted of 71.8% followed by ceftriaxone monotherapy. EATs were converted to others in 20 (14.8%) and 2 (1.5%) patients for the first and second times respectively, mainly based on poor clinical response without conducting culture and sensitivity tests. These tests were done only for 13 (9.6%) participants and growth was seen in 5 patients; and definitive therapy was given for 2 patients. ANC value was above 500 cell/mm^3^ in 80.7% of patients and 98.5% of study participants were afebrile after completion FN treatment. Most of them (70.4%) were treated for FN and 7 of patients died due to all case mortality. The hospital should not rely mainly only on ceftriaxone with gentamycin as EAT and should do culture and sensitivity test to optimize therapy based on susceptibility result before conversion and modification of therapy in management of FN.

## Introduction

Febrile neutropenia (FN) is an oral temperature of > 38.3 °C or two consecutive readings of > 38.0 °C for 2 h and an absolute neutrophil count (ANC) of < 0.5 × 10^9^/l, or expected to fall below 0.5 × 10^9^/l [1]. In cancer patients, it is a serious, potentially fatal condition complicating cancer treatment associated with significant morbidity and mortality [1, 2] and common reasons for hospital admission in children with cancer [3, 4]. Factors which contribute to occurrence of FN in pediatric cancer patients include age of child, duration of neutropenia [4], ANC value, co-morbidities, type of cancer and its stage, complication of chemotherapy [5], identified pathogens, the severity of vital sign [6].

The classification of FN in terms of the risk of infection helps to choose antimicrobial therapy, in defining the route of administration, the possibility of outpatient treatment and the probable etiology [7]. National Comprehensive Cancer Network guideline stratifies patients according to Multinational Association for Supportive Care in Cancer (MASCC) index to categorize them to the low or high risk. According to the MASCC scores, a patient with a score < 26 is to be considered at low risk and treated as an outpatient, whereas a patient with a score > 21 is at high risk and should be hospitalized [1].

Empiric broad-spectrum antibiotic therapy is the main management initiated for the treatment of FN in patients with cancer [5, 7]. Guidelines for the management of FN in cancer patients have continued to grow, but still include broad-spectrum antibiotic therapy at the onset to reduce the mortality rate that is associated with a delayed treatment [8, 9]. In addition to empiric antibiotics therapy (EAT), the management of FN in cancer patients includes a thorough physical examination, blood cultures, including sampling from all lumens of a central venous line if present, and other investigations depending on presenting signs and symptoms local antibiotic resistance patterns [7, 8].

In Ethiopia, there is no standard protocol established for the management of FN in pediatric patients with cancer. Furthermore, sufficient studies are not available regarding management of FN in this patient population. Thus, this study assessed the gap that might exist in management of FN in pediatric patients admitted to the pediatric oncology ward of a largest teaching tertiary hospital in the country.

## Main text

### Methods

#### Study setting

This study conducted in pediatric oncology ward of Tikur Anbessa Specialized Hospital (TASH) on pediatric patients with cancer. TASH is the largest tertiary university teaching hospital found in Addis Ababa, Ethiopia with bed capacity of 570 beds and out of which 130 allocated for pediatric patients. Pediatric oncology ward contains 26 beds and around nearly 200 children with cancer are admitted yearly in this ward. This hospital selected for this study as it is the main referral center in the country in which patients who have different cancer are managed since many years.

#### Study design and period

A retrospective cross-sectional used to collect data from patients’ charts among those admitted from January 1, 2017 to December 31, 2017 (1 year) using data abstraction tool in pediatric patients with cancer.

### Source and study population

#### Study participants

All pediatric patients admitted with cancer at pediatric oncology ward of the hospital and cancer patients who developed FN after admission to this ward are source and study population in this study, respectively. Pediatric patients with cancer whose age ≤ 15 years with diagnosis of FN and those who stayed in the hospital at least 2 days were included in this study. However, we excluded patients who readmitted with recurrent FN and charts with ineligible handwriting which was difficult to extract patients’ information.

#### Sample size determination

All patients admitted to pediatric oncology ward of hospital from January 1, 2017 to December 31, 2017 and accordingly 135 study participants met inclusion criteria were included in our study.

#### Data collection, management and quality assurance

We collected data from patients’ charts using structured data abstraction tool. It contained detail information on patients demographics (weight, height, age, sex, hospital stay), clinical data (diagnosis, type of hematologic malignancy, co-morbidities, ANC values, body temperature measurements, culture and sensitivity test results, selected empiric and definite therapy for FN including name of the drug, dose, frequency duration of therapy (starting and ending date) and treatment modifications. Training was provided to data collectors before starting data collection to maintain quality of the data. During data collection process, the principal investigator was closely followed data collectors activities. Data was entered into a computer using Epi-info 7 and exported to Statistical Program for Social Sciences (SPSS) version 20 for analysis. Data was described in percentage, frequency and mean ± SD when necessary.

#### Ethical considerations

Ethical clearance and approval obtained from the Ethical Review Board of School of Pharmacy, Addis Ababa University. Then, official support letters written by Department of Pharmacology and Clinical Pharmacy to TASH and permission obtained from the hospital to conduct the study.

### Results

#### Socio-demographic and clinical characteristics

Even if, 532 patients admitted to pediatric oncology ward of hospital during the stated period, we could not get many patients’ charts that fulfilled inclusion criteria. Hence, finally 135 patients were included in final analysis.

In our study, nearly more than half of participants (54.1%) were aged between > 1 years to ≤ 5 years with mean age of 5.5 (SD = 3.2) years. Out of 135 study participants, more than two-third (68.9%) were male patients. Acute lymphocytic leukemia was diagnosed in 52.6% of study participants followed by different types of blastomas (Table 1).

**Table 1:** Socio-demographic and clinical characteristics of the patients at pediatric oncology ward of TASH (N = 135)

Variables	Category	N (%)
Age	≤ 1 years	7 (5.2)
	> 1 year to ≤ 5 years	73 (54.1)
	> 5 years to ≤ 10 years	41 (30.4)
	> 10 years to ≤ 15 years	12 (8.9)
Sex	Male	93 (68.9)
	Female	42 (31.1)
Type of malignancy	Acute lymphocytic leukemia	71 (52.6)
	Acute myeloid leukemia	10 (7.4)
	Non-Hodgkin lymphoma	10 (7.4)
	Blastomas	17 (12.6)
	Sarcomas	15 (11.1)
	Others^a^	12 (8.9)
Presence of co morbid illness	Hypertension	10 (7.4)
	Congestive heart failure	6 (4.4)
	Tuberculosis	1 (0.7)
	Others^b^	9 (6.7)
ANC value before starting treatment (cell/mm^3^)	< 500	120 (88.9)
	500–1000	9 (6.7)
	1000–1500	5 (3.7)
	> 1500	1 (0.7)
Focus of neutropenic infection	Chest focus	15 (11.1)
	Gastro intestinal focus	5 (3.7)
	Urinary focus	2 (1.5)
	Unknown focus	113 (83.7)
Patients fulfillment FN diagnostic criteria	Yes	116 (85.9)
	No	19 (14.1)
FN diagnosed	Before initiation of chemotherapy	47 (34.8)
	After chemotherapy	88 (65.2)

^a^Hodgkin lymphoma, Wilms tumor, Brain and spinal cord tumors

^b^Gastric infection, HIV, diarrhea, malnutrition

Among the study population, 116 (85.9%) fulfilled the FN diagnostic criteria and in the remaining diagnosis was made without evidence and 88.9% patients had ANC value of less than 500 cell/mm^3^ before starting antibiotic therapy. Focus of FN infection was unknown in most of (83.7%) of study participants (Table 1).

#### Empiric antibiotics therapy and treatment conversion practice in management of FN

Empiric antibiotics therapy (EAT) was given to all patients in which ceftriaxone with gentamycin constituted 71.8% and followed by ceftriaxone monotherapy. The mean duration of treatment for FN was 9 (SD = 5) days. Majority of patients took EAT for 7–14 days of treatment (Table 2).

**Table 2:** Empiric antibiotics therapy utilization and conversion practice in management of FN in pediatric oncology ward of TASH (N = 135)

Item description	N (%)
Empiric antibiotics therapy indicated	
Ceftriaxone + gentamycin	97 (71.8)
Cefepime + gentamycin	4 (3.0)
Ceftazidime + vancomycin	3 (2.2)
Ceftriaxone	6 (4.4)
Ceftriaxone + vancomycin	5 (3.7)
Cefepime + vancomycin	4 (3.0)
Meropenem + ciprofloxacin	2 (1.5)
Meropenem + gentamycin	1 (0.7)
Piperacilin/tazobactam	4 (3.0)
Meropenem + vancomycin	4 (3.0)
Others^a^	5 (3.7)
Duration of empiric antibiotics therapy	
< 7 days	60 (44.4)
> 7 to ≤ 14 days	66 (48.9)
> 14 days	8 (5.9)
Antibiotic conversion status	
First time conversion	20 (14.8)
Second time conversion	2 (1.5)
First time conversion	
Monotherapy	3 (2.2)
Combination therapy	17 (12.6)
Second time conversion	
Monotherapy	1 (0.7)
Combination therapy	1 (0.7)

^a^Includes ampicillin/sulbactam, cefepime + vancomycin + gentamycin, meropenem + gentamycin, meropenem + ciprofloxacin + vancomycin,piperacilin/tazobactam + vancomycin + ciprofloxacin, and ceftraxone + vancomycin. Duration of EAT and antibiotics conversation status couldn’t add to 10% due to 1 missing data and only in 16.3% conversation was made, respectively

EATs were converted to others in 20 (14.8%) and 2 (1.5%) cases for the first and second times respectively, mainly based on poor clinical response and without conducting culture and sensitivity tests.

#### Definitive therapy and antibiotics addition practice and FN related outcomes

Culture and sensitivity was done only for 13 (9.6%) participants. Out of these tests, 3 and 10 tests were done before and after starting antibiotics respectively. Bacterial growth was seen in 5 of them, and however, definitive therapy was given only 2 for patients. Other than main antibiotics (EAT and those converted), additional antibiotics (both prophylaxis and treatment) were added in 57 (42.2%) patients based on response to main antibiotics indicated for management of FN (Table 3).

**Table 3:** Additional antibiotics and neutropenic fever related outcomes in management of neutropenic fever in pediatric oncology ward of TASH (N = 135)

Item description	N (%)
Antiviral added	
Yes	30 (22.2)
Acyclovir	30 (22.2)
No	105 (77.8)
Antifungal added	
Yes	52 (38.5)
Fluconazole	43 (31.8)
Itraconazole	1 (0.7)
Amphotercin B	1 (0.7)
Miconazole	7 (5.2)
Other^a^	7 (5.2)
No	83 (61.5)
Antibacterial added	
Yes	15 (11.1)
Ciprofloxacin	1 (0.7)
Cotrimoxazole	9 (6.7)
Cotrimoxazole + ciprofloxacin	5 (3.7)
No	120 (88.9)
Antibiotic finished place	
In the hospital	118 (87.4)
Outside of the hospital	17 (12.6)
ANC value after antibiotic treatment in cell/mm^3^	
< 500	26 (19.3)
500–1000	33 (24.4)
1000–1500	29 (21.5)
> 1500	47 (34.8)
Body temperature after FN treatment	
Afebrile	133 (98.5)
Febrile	2 (1.5)
Outcome related to FN	
Treated	95 (70.4)
All cause mortality	7 (5.2)
Unknown status	33 (24.4)

^a^The percentage of antibiotics addition couldn’t add up to hundred as a patient can receive antiviral, antifungal and antibacterial at the same time

Most of patients (87.4%) finished their antibiotics in the hospital. About 80% of study participants had ANC value of ≥ 500 cell/mm^3^ and almost all of them were afebrile when they finished FN therapy. Most of them (70.4%) were treated for FN and 7 of patients died due to all case mortality (Table 3).

### Discussion

Febrile neutropenia is majorly associated with the treatment and the type of the cancer diagnosis [1]. Despite major advances in prevention and treatment, FN remains one of the most frequent and serious complications of cancer chemotherapy. Still, no single algorithm adopted for use in pediatric patients with FN. Therefore, the current study aimed to assess the management practice of febrile neutropenia in childhood cancer patients.

In the present study, FN was common in the age group of 1–5 years 73 (54%). This is in fact that most of the acute leukemias cases are encountered in this age group, which makes them vulnerable for neutropenic fever [10]. In our study, acute lymphocytic leukemia (ALL) was the most frequent diagnosed cancer type among febrile neutropenic patients in more than half of the participants. Similarly a study done in Pakistan showed that ALL was the most frequent underlying malignancy (84.3%) and the highest FN episodes (72.1%) occurred during the induction phase treatment [11]. Our finding showed that acute leukemias constituted 60% of the cancer diagnosis followed by Non-Hodgkin lymphoma (7.4%) and solid tumors. In line with the present study, a study from Mexico reported acute leukemias were the most types (65.2%) of the cancer diagnosis followed by lymphomas (14.1%) and solid tumors in pediatrics [12].

In our study, most of patients (85.9%) fulfilled the criteria of diagnosing and treating FN. However, in some patients, treatment started irrespective of the neutropenia criteria. This was seen mainly in acute myeloid leukemia since majority of the neutrophils in this type of cancer are dysfunctional which leads patients at high risk of infection [13].

Regarding to the EAT, combination of ceftriaxone and gentamycin constituted around 72% of the regimen in the current study. But comparative study at Bangabandhu Sheikh Mujib Medical University Hospital showed that empirical treatment of FN with combined cefepime and amikacin is more effective than combination of ceftriaxone and gentamicin in children with malignancies [14]. Empiric antibiotic therapy with agents active on viridans group Streptococci and *Pseudomonas aeruginosa* are recommended as a standard of care in high-risk FN childhood cancer patients to provide coverage for virulent organisms while minimizing exposure to unnecessary antibiotics, because indiscriminant use of broad-spectrum antibiotics may accelerate antibiotic resistance rates [3, 15]. β-Lactams and aminoglycosides were the most common antibiotics used in our setup. Similar finding was reported by Llamas et al. as 75.5% of patients were received combination of a β-lactam with an aminoglycoside [11]. Other study done by Vathana et al. also showed that, 77.1% of patients were treated with a combination of cefatazidime and gentamycin [16]. In present study, the mean duration FN treatment was 9 days which is lower than report from two Mexican studies [11, 17].

In this study, culture and sensitivity was done only for 13 (9.6%) participants and out of which 3 and 10 tests were taken before and after starting EAT, respectively. In consistent with our study, a study conducted by Llamas et al. showed that blood culture was taken in only 13.5% of the study subjects and majority (71.4%) of the samples were collected after the initiation of EAT [11]. This high inappropriate sample collection time in our study might be due to most patients come to our hospital after exposed to many drugs in lower level health facilities and low awareness of the health care professionals towards culture and susceptibility test.

In the present study, additional antiviral, antifungal and antibacterial added for some patients. Addition of antifungal agent is recommended for patients with neutropenia who remains febrile for 4–7 days after broad-spectrum antibiotic therapy or recurrent fever with persistent neutropenia [18, 19]. Study from Switzerland reported that antifungals were added in 4 (9%) unresolved FN episodes [20].

In our study, antibiotics therapy was discontinued in 19.3% of the participants without clearly documenting the reasons(s) behind that. The great discrepancy in our study might be due to the absence of local guideline for the initiation, modification and discontinuation of EAT. All cause mortality was observed in 7 (5.2%) of the study participants. This is in lined with a study conducted in Pakistan which showed that death was occurred in 7 (5.2%) [12].

### Conclusion

All patients received EAT for management of FN. Ceftiaxone with gentamycin is the most combination EAT used for management of FN in study population. The hospital shouldn’t rely mainly only on ceftriaxone with gentamycin as EAT and should do culture and sensitivity test to optimize therapy based on susceptibility result before conversion and modification of therapy.

## Limitations of study

We used smaller sample size which might have influence on some of the results reported in this study. Apart from this, the data retrieval was based on the written information in medical records, which might be confounded by physicians’ difference in documentation of patients’ related data and thus may not reflect the real practice in some occasions.

## Data Availability

All data used and/or analyzed during this study are included in this manuscript study are and available from the corresponding author on request.

## Abbreviations

ANC: absolute neutrophil count
ALL: acute lymphocytic leukemia
EAT: empiric antibiotic therapy
FN: febrile neutropenia
MASCC: Multinational Association for Supportive Care in Cancer
SD: standard deviation
SPSS: Statistical Package for Social Science
TASH: Tikur Anbessa Specialized Hospital
